# Efficient IoT User Authentication Protocol with Semi-Trusted Servers

**DOI:** 10.3390/s25072013

**Published:** 2025-03-23

**Authors:** Shunfang Hu, Yuanyuan Zhang, Yanru Guo, Wang Zhong, Yanru Chen, Liangyin Chen

**Affiliations:** College of Computer Science, Sichuan University, Chengdu 610065, China; hsf@ymu.edu.cn (S.H.); yuanyuanzhang@stu.scu.edu.cn (Y.Z.); guoyanru@stu.scu.edu.cn (Y.G.); chenyanru@scu.edu.cn (Y.C.)

**Keywords:** internet of things, user authentication, insider attacks, key privacy, semi-trusted servers

## Abstract

Internet of Things (IoT) user authentication protocols enable secure authentication and session key negotiation between users and IoT devices via an intermediate server, allowing users to access sensor data or control devices remotely. However, the existing IoT user authentication schemes often assume that the servers (registration center and intermediate servers) are fully trusted, overlooking the potential risk of insider attackers. Moreover, most of the existing schemes lack critical security properties, such as resistance to ephemeral secret leakage attacks and offline password guessing attacks, and they are unable to provide perfect forward security. Furthermore, with the rapid growth regarding IoT devices, the servers must manage a large number of users and device connections, making the performance of the authentication scheme heavily reliant on the server’s computational capacity, thereby impacting the system’s scalability and efficiency. The design of security protocols is based on the underlying security model, and the current IoT user authentication models fail to cover crucial threats like insider attacks and ephemeral secret leakage. To overcome these limitations, we propose a new security model, IoT-3eCK, which assumes semi-trusted servers and strengthens the adversary model to better meet the IoT authentication requirements. Based on this model, we design an efficient protocol that ensures user passwords, biometric data, and long-term keys are protected from insider users during registration, mitigating insider attacks. The protocol also integrates dynamic pseudo-identity anonymous authentication and ECC key exchange to satisfy the security properties. The performance analysis shows that, compared to the existing schemes, the new protocol reduces the communication costs by over 23% and the computational overhead by more than 22%, with a particularly significant reduction of over 95% in the computational overhead at the intermediate server. Furthermore, the security of the protocol is rigorously demonstrated using the random oracle model and verified with automated tools, further confirming its security and reliability.

## 1. Introduction

**Figure 1:**
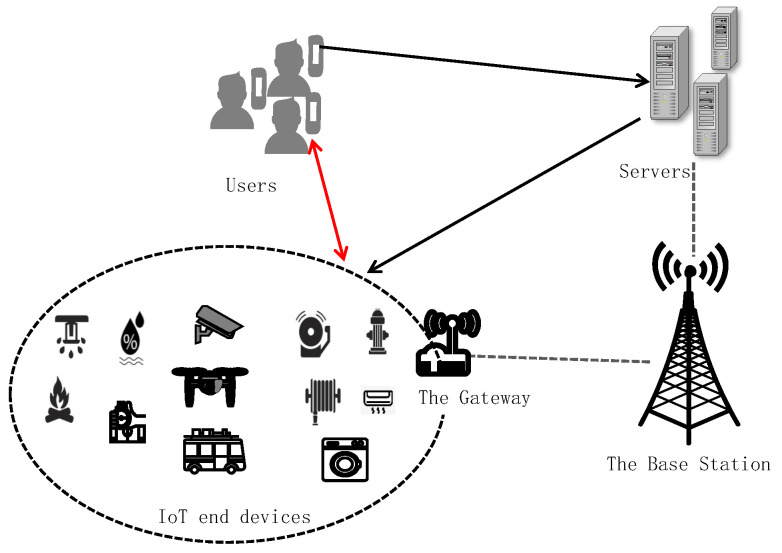
Structure of IoT systems.

The Internet of Things (IoT) is transforming the way we interact with the physical world by connecting everyday devices to the Internet, creating an intelligent and automated ecosystem [1]. The IoT is transforming multiple fields, including industry, agriculture, power grids, transportation, the environment, healthcare, smart homes, and security and surveillance. As illustrated in Figure 1, the IoT system is composed of IoT devices, gateways, servers, and remote users, which form the foundation to enable the acquisition, processing, and transmission of real-time data. In smart homes, smart healthcare, and industrial IoT application systems, users need to remotely access or control IoT devices and send them specific instructions [2]. Without effective user authentication, attackers can impersonate legitimate users and send fake malicious instructions to IoT devices, threatening the security of the entire system. IoT user authentication protocols enable secure authentication and session key negotiation between users and IoT devices through an intermediate server, allowing users to access or control IoT devices directly and securely. Therefore, establishing a robust IoT authentication mechanism is crucial to ensuring these systems’ security [3].

**Figure 2:**
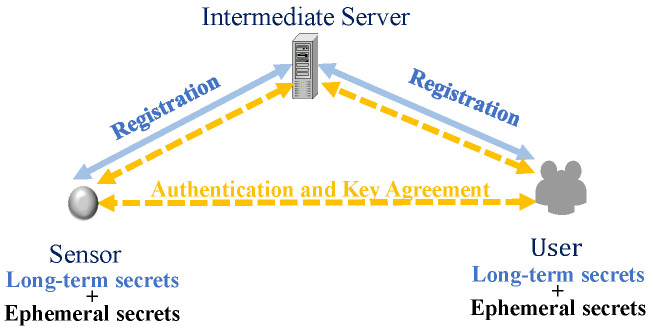
IoT user authentication protocol.

As illustrated in Figure 2, in the IoT user authentication protocols, after the devices and users register, each entity obtains its own long-term key. Subsequently, the user and the IoT device use these long-term keys, as well as the temporary keys generated during authentication, to generate authentication messages. These messages are then exchanged between the two entities for mutual authentication and negotiation of session keys. However, IoT devices are easily captured physically when they are usually deployed in unattended environments. Attackers may gain access to long-term secrets through side-channel attacks [4,5]. In addition, servers may suffer from data leakage and unauthorized access due to malware or misconfigurations [6,7]. Given that IoT systems often involve sensitive data, including user location information, network activities, and consumption habits, as well as potentially confidential corporate or national security information, authentication and authorized data access have become essential security requirements for IoT applications. In addition, the design of authentication protocols is particularly challenging as IoT devices are resource-limited, usually powered by batteries, and have limited computing, storage, and communication capabilities [8].

In recent years, numerous IoT user authentication protocols [9,10,11,12,13,14,15,16,17,18,19,20,21,22,23,24,25,26] have been proposed as shown in Table 1. However, the existing schemes generally assume that the servers (registration center (RC) and intermediate server) are fully trusted, overlooking the potential threat of insider attackers. In real applications, server data breaches and unauthorized port listening events are common due to issues such as misconfigurations, memory leaks, and malware. In addition, insider-infected actions (such as employees, contractors, or partners) pose significant security risks to IoT systems [6,7]. During registration, the RC generates long-term keys for the IoT devices and stores them on both the devices and the intermediate server. This setup allows insiders on the servers to access the keys, potentially enabling insider attacks. For example, insider users on the intermediate server can perform insider impersonation attacks [9,10,12,13,14,15,17,19,22,23,24,25,26] or derive session keys negotiated between users and IoT devices, resulting in protocols with no key privacy security [9,10,12,13,15,26]. Another critical issue is the handling of ephemeral secrets, which are often precalculated and stored in insecure memory [27]. Unfortunately, this risk is largely neglected in current IoT user authentication protocols [9,10,12,13,14,15,16,17,18,19,20,21,22,24,25,26], leaving these schemes vulnerable to ephemeral secret leakage (ESL) attacks. Furthermore, many protocols lack essential features, such as perfect forward security (PFS) and anonymity. They are also prone to offline password guessing attacks and are inefficient for resource-constrained IoT devices. More importantly, with the rapid growth regarding IoT devices, intermediate servers must manage a large number of users and device connections, which makes the performance of authentication schemes highly dependent on the server’s computational and processing capabilities [25]. However, there is still a lack of substantial research focused on optimizing server overhead in the current studies.

The design of security protocols is based on the underlying security model, where the attacker model defines the potential capabilities and goals of the attackers. This model not only determines the security assumptions of the protocol but also directly shapes the evaluation framework for security analysis. In the existing research, the Dolev–Yao security model [29] and the eCK security model [30] are the most widely accepted. The Dolev–Yao model assumes that the attacker has full control over the communication channel, allowing him to intercept, modify, forge, and replay messages. The eCK model extends this by allowing the attacker to access sensitive information, such as long-term keys, session keys, and ephemeral secrets. However, the eCK model focuses mainly on two-party single-factor authentication protocols and does not fully address multifactor or three-party authentication scenarios. In recent years, there has been growing attention regarding attacker models for multifactor user authentication protocols. Yoneyama [31] extended the eCK model to password-based three-party authentication key-exchange protocols but did not account for threats related to smart card loss and offline password guessing attacks. Wang et al. [32] studied the impact of the loss of smart cards and offline password guessing attacks on the security of two-factor authentication protocols, and their results have been adopted as a standard in many subsequent schemes. However, their work did not address the leakage of ephemeral secrets. In conclusion, the existing security models for IoT user authentication fail to fully cover key security threats, such as insider attacks and ephemeral secrets.

To address the issues in the existing IoT user authentication protocols, we introduce a new security model, IoT-3eCK. This model is based on real-world network threats, enhances the attacker’s capabilities, and refines the evaluation criteria for IoT user authentication protocols. In the IoT-3eCK model, the servers are assumed to be semi-trusted entities, and the attackers may be insiders from either the RC or the intermediate server. The attacker has access to the registration information of users, servers, and IoT devices and can acquire the ephemeral secrets of all the protocol participants, including users, servers, and IoT devices. Based on this new security model, we analyze the Wang et al. scheme [25], identifying the challenges posed by insider and ESL attacks and uncovering the causes of their vulnerabilities. Then, we propose a new IoT user authentication protocol that effectively defends against insider attacks while meeting various security requirements. The protocol integrates a dynamic pseudonym-based anonymous authentication mechanism with an ECC key-exchange mechanism, ensuring anonymity and other critical security properties while significantly reducing the computational overhead on the intermediate server. The performance analysis shows that, compared to the existing solutions, the proposed protocol reduces communication overhead by more than 23%, computational overhead by over 22%, and, most notably, computational overhead on the intermediate server by over 95%, greatly improving the system efficiency.

The main contributions of this paper are as follows.

(1) A new security model, IoT-3eCK, is proposed, which enhances the attacker’s capabilities regarding IoT user authentication protocols and refines the evaluation criteria for these protocols.
(2) Based on the new security model, analysis of the Wang et al. scheme [25] is provided, identifying the challenges of insider and ESL attacks and uncovering the causes of their vulnerabilities.
(3) A secure and efficient IoT user authentication protocol is introduced. The proposed scheme offers security properties such as key privacy and perfect forward security and is resilient to insider attacks, ESL attacks, and other common threats.
(4) The security of the protocol is formally proven using the random oracle model, and its correctness is verified with the ProVerif 2.0 automated verification tool. In addition, performance comparisons demonstrate significant reductions in communication and computation costs.

The paper is structured as follows. Section 2 reviews the related work. Section 3 presents the proposed new security model. Section 4 provides a cryptanalysis of the scheme by Wang et al. [25]. Section 5 outlines the proposed IoT user authentication protocol. Section 6, Section 7, Section 8 and Section 9 present the security analysis and performance comparison, respectively. Finally, Section 10 concludes the paper.

## 2. Related Work

With low computational complexity and high efficiency, many user authentication protocols based on symmetric cryptography [33,34,35] have been proposed. However, these proposals require users and designated IoT devices to share symmetric keys beforehand, which is impractical due to the large number of IoT devices. Physically Unclonable Functions (PUFs), a promising lightweight hardware security primitive, have been used in numerous user authentication protocols for the IoT [3,36,37]. In these schemes, each entity needs to register one or more challenge–response pairs of its individual PUF with the server beforehand to enable authentication between the registered entity and the server, which leads to inflexibility and inefficiency. Halevi et al. [38] and Wang et al. [39] also indicated that most symmetric cryptography and hash-based user authentication proposals are insecure when dealing with user anonymity, PFS, and smart card security breach attacks. Public-key technology is vital for improving security, but it has high computational and communication overhead. Elliptic curve cryptography (ECC) has been adopted in IoT user authentication to balance security and efficiency because it provides a smaller key size compared to other methods with the same level of security [40,41].

In recent years, many ECC-based IoT user authentication protocols have been introduced. In 2016, Jiang et al. [9] introduced a user authentication protocol for wireless sensor networks (WSNs), a critical component of the IoT. This protocol employs two-factor authentication through temporary credentials and claims to resist various attacks, ensuring both the anonymity and untraceability of communications. However, the protocol assumes that the servers are fully trusted, neglecting the security threat posed by insider attackers within the servers. During registration, the RC generates and preloads long-term secrets for the sensors, storing these credentials on the intermediate server. This design exposes the protocol to insider impersonation attacks and key privacy issues, where insider users of the servers can impersonate sensors to complete authentication and key agreement with users, as well as obtain the session key negotiated between the user and the sensor during the authentication process. In addition, the scheme does not account for the possibility that an attacker may gain access to the ephemeral secret, making it vulnerable to ESL attacks. Furthermore, the scheme is also susceptible to offline password guessing attacks and does not provide PFS and timely typo detection. In 2017, Wu et al. [10] indicated that Chang et al. [11] were unable to resist offline password guessing attacks and could not maintain PFS. Then, they presented an improved one for WSNs, but it lacks user untraceability, key privacy, and PFS and suffers from offline password guessing, ESL, and insider sensor impersonation attacks. Challa et al. [12] introduced signature-based user authentication for the IoT. However, it is incapable of withstanding attacks of offline password guessing, impersonation, and insider IoT device impersonation, as well as failing to provide key privacy, user anonymity, and untraceability. In 2018, Li et al. [13] presented an IoT user authentication protocol for industrial IoT to ensure legitimate access to sensitive sensor data. But, this proposal [13] suffers from offline password guessing, replay, insider sensor impersonation, and impersonation attacks and lacks key privacy. Li et al. [14] identified the deficiencies of the scheme [9] and then presented an enhanced scheme for WSNs. However, the enhanced scheme [14] is prone to offline password guessing, insider sensor impersonation, and ESL attacks and cannot offer user untraceability, key privacy, and PFS. In 2019, Shuai et al. [15] presented an IoT user authentication scheme for smart homes. However, this scheme is subject to offline password guessing, insider IoT device impersonation, and ESL attacks and is unable to offer user untraceability, key privacy, and PFS. Lu et al. [28] introduced an IoT user authentication scheme for WSNs. Moreover, this scheme had a design flaw: i.e., an attacker could quickly determine the session key based on the sensor ID and the information detected throughout the open channels. In 2020, Li et al. [17] stated that most of the existing AKA proposals could not fulfill local password change and PFS while being vulnerable to stolen smart card attacks, and then introduced an improved version. Unfortunately, the scheme [17] is prone to sensor impersonation and offline password guessing attacks with poor usability. The server must enumerate the stored information and then calculate the relevant values to determine the sender’s ID when a reply message is received during authentication.

In 2021, Sadhukhan et al. [18] presented an authentication scheme for remote IoT users, but it suffers from replay, ESL, man-in-the-middle, and DoS attacks and cannot offer user anonymity and timely typo detection. Wazid et al. [19] introduced an IoT user authentication protocol to secure 6G-enabled networks in a box deployed in industrial applications. Wang et al. [25] revealed that the scheme [19] fails to provide user untraceability and PFS; it is also vulnerable to offline password guessing, desynchronization, and man-in-the-middle attacks. Additionally, the scheme lacks key privacy and cannot withstand insider IoT device impersonation attacks. Srinivas et al. [20] proposed an IoT user authentication scheme for IoT big data collection. However, this scheme [20] is fragile to device impersonation attacks and device session key compromise attacks and cannot afford user anonymity and PFS [22]. In 2022, Sutrala et al. [21] introduced an IoT user authentication scheme for software-defined network-based industrial cyberphysical systems [21]. Later, Wang et al. [23] stated that the scheme [21] is not able to resist smart card/device loss attacks and offline password guessing attacks and cannot offer user untraceability. Tanveer et al. [22] analyzed previous related IoT user authentication protocols and then proposed an improved version for the Internet of Drones. However, the scheme [22] cannot maintain user untraceability, PFS, or resistance to man-in-the-middle attacks and offline password guessing attacks. In 2023, Chen et al. [24] introduced a two-factor user authentication protocol for multi-gateway WSNs. Unfortunately, this scheme suffers from insider sensor impersonation and ESL attacks. Wang et al. [25] analyzed Wazid et al. [19] and then proposed an IoT user authentication scheme for cloud-assisted IoT with four parties prone to insider IoT device impersonation and ESL attacks. In 2024, Kumar et al. [26] proposed an IoT user authentication scheme to access sensitive data from sensor nodes by executives in coal mining scenarios. But, this scheme is not resistant to insider sensor impersonation attacks and ESL attacks, lacking PFS and key privacy.

In summary, the previous IoT user authentication schemes suffered from insider sensor impersonation attacks. Furthermore, most of the schemes are vulnerable to ESL and offline password guessing attacks, lack essential security features such as PFS, key privacy, and anonymity, or are unsuitable for resource-constrained IoT due to inefficiencies.

## 3. Security Model Based on Semi-Trusted Servers

This section defines a practical security model, IoT-3eCK, based on the network model. The model enhances the attacker’s capabilities and introduces an evaluation framework consisting of 10 attributes and 12 security goals, which supports a comprehensive analysis of IoT user authentication schemes.

### 3.1. IoT User Authentication Network Model

As illustrated in Figure 1, the network model consists of remote users, intermediate servers, IoT devices, and adversaries. RCs are specialized servers that are responsible for overseeing key generation, distribution, management, and communication activities within an application system or group. We assume that an IoT user authentication protocol, P, involves $U_{i}\left(1\leq i\leq r\right),S_{j}\left(1\leq j\leq s\right)$ and $S_{k}\left(1\leq k\leq t\right)$, where r≫s,t≫s, *r*, *s*, and *t* denote the number of users, servers, and IoT devices, respectively. The servers are considered semi-trusted, meaning they will reliably execute the protocol but may also collaborate with other participants to potentially gain private information. Each remote user can dynamically join different systems or groups by registering with a different RC. However, a sensor can belong to a single system or group.

### 3.2. Adversary Model

To characterize the insider threat, we introduce the attacker capability A7: assume that A is an insider user of RC, capable of corrupting servers, learning the long-term private key of the servers, and eavesdropping on and stealing messages during the registration, login, and authentication phases. To address the threat of ephemeral key disclosure, we introduce the attacker capability A8: assume that A can obtain ephemeral secrets of the user, sensor, and RC. The primary capabilities of A in the new adversary model, IoT-3eCK, for the IoT user authentication protocol are as follows:

A1. A takes full control over communication channels, intercepting, modifying, blocking, and deleting messages exchanged between the user, the RC, and the IoT devices at will.
A2. A can offline all items in the set $D_{id}\times D_{pw}$ of the space $D_{id}$ and password space $D_{pw}$ within polynomial time.
A3. A is able to capture the previous session keys between the user and the sensor.
A4. A can learn the user’s passwords, extract long-term secrets from the smart card, and obtain the user’s biometrics, but not all three at the same time.
A5. A is able to compromise the sensor and extract its long-term secrets.
A6. A can obtain the secret key of RC when evaluating forward secrecy.
A7. The adversary A could be an insider user of the RC or compromise the server *S*, gaining access to its long-term private key and intercepting/stalling messages during any phase (registration, log-in, authentication).
A8. A can obtain ephemeral secrets of the user, sensor, and RC.

### 3.3. Evaluation Criteria

Our evaluation criteria are modified from widely accepted frameworks [2,8,25,42,43]. To mitigate insider attacks, C1 and C2 are introduced. First, during the registration phase, the authentication secrets must be protected, ensuring that the long-term secrets of the user and sensor, as well as the user’s password and biometric data, are not accessible to insider users of the RC. Second, insider users of intermediate servers, including those in the RC, should not have access to the session keys established between the user and the sensor. Furthermore, C10 has been improved to include resistance to ESL attacks and insider impersonation attacks, further strengthening its defenses against known threats.

C1. No registering confidential exposure: When registering the long-term secrets of the user and sensor, the user’s password and biometrics cannot be derived by the insider user of RC.
C2. Key privacy: The insider user of RC or the intermediate server cannot access the session keys between the user and the sensor.
C3. No password verifier-table: RC does not store user passwords or any derived password values in a database.
C4. Password-friendly: The user should freely choose and change an easy-to-remember password locally.
C5. Sound repairability: The scheme enables the easy revocation of smart cards, that is, the users are able to revoke their cards with unchanging identity.
C6. Mutual authentication and key agreement: The user and the sensor can verify each other and agree on a shared session key.
C7. Forward secrecy: The shared session key could not be captured even if the adversary obtained the long-term secrets of the user, the RC, and the sensor.
C8. Timely typo detection: If a user enters an incorrect password or biometrics when logging in, he or she will be informed in time.
C9. User anonymity: The scheme is designed to protect user identity and prevent user activities from being traced.
C10. Resistance to known attacks: The scheme resists known attacks, including ESL attacks, insider impersonation attacks, unknown key share attacks, known key attacks, smart card loss attacks, stolen verifier attacks, impersonation attacks, desynchronization attacks, parallel session attacks, replay attacks, offline password guessing attacks, key control, and node capture attack.

## 4. Analysis of Wang et al.’s Protocol

Wang et al. [25] proposed a secure and efficient IoT user authentication protocol for cloud-assisted IoT systems. Participants include IoT devices, gateways, cloud servers, and external users. Security analysis concluded that this scheme is vulnerable to insider impersonation attacks and ESL attacks.

### 4.1. Review of Wang et al.’s Protocol

The notations used are listed in Table 2.

#### 4.1.1. Registration Phase

In the registration phase, the IoT device is registered with the gateway, and both the gateway and the user are registered with the cloud server. The cloud server first selects an elliptical curve *E*, with a base point *P*. After initialization, the cloud server possesses long-term secret keys *x* and *y*(Y=y·P), shared with the user and the gateway, respectively. The cloud server distributes the gateway secret key $X_{G_{k}}=h\left(x∥GID_{G_{k}}\right)$ to the gateway as an authentication credential. The gateway and the IoT devices $S_{k}$ share a secret key $X_{sk}=h\left(ID_{sk}∥x_{G_{k}}\right)$, where $x_{G_{k}}$ is the long-term secret key of the gateway.

The user $U_{i}$ registration process is as follows:

R1. $U_{i}$ first selects the identity $ID_{ui}$ and password $PW_{ui}$, and inputs the biometric factor $Bio_{ui}$. Then, $U_{i}$ selects a random number $a^{\prime }$ and calculates $Gen\left(Bio_{ui}\right)=\left(\delta_{ui},\tau_{ui}\right)$ and $RPW_{ui}=h(PW_{ui}∥\delta_{ui}∥a^{\prime })$. Next, $U_{i}$ sends the registration request $\left\{ID_{ui},RPW_{ui}\right\}$ to the cloud server.
R2. The cloud server selects a timestamp $T_{rgui}$ and a random number $a_{ui}$ and then calculates $k_{ui}=h(ID_{ui}∥y∥T_{rgui})$ and $B_{ui}^{\prime }=h\left(RPW_{ui}∥ID_{ui}\right)⊕k_{ui}$. The cloud server stores $\left\{ID_{ui},T_{rgui},a_{ui},Honey-list=NULL\right\}$ in the database and sends $\left\{B_{ui}^{\prime },B_{ui}^{\prime }⊕a_{ui},Y,P\right\}$ to $U_{i}$.
R3. Upon receiving $\left\{B_{ui}^{\prime },B_{ui}^{\prime }⊕a_{ui},Y,P\right\}$, $U_{i}$ selects a random number *a* and then calculates $k_{ui}=B_{ui}^{\prime }⊕h\left(RPW_{ui}∥ID_{ui}\right)$, $RPW_{ui}^{new}=h(PW_{ui}∥\delta_{ui}∥a)$, $A_{i}=h(ID_{ui}∥RPW_{ui}^{new}∥k_{ui})\;\mathrm{mod}\;n_{0}$, $B_{ui}=h\left(RPW_{ui}^{new}∥ID_{ui}\right)⊕k_{ui}$, $a_{ui}=B_{ui}^{\prime }⊕\left(B_{ui}^{\prime }⊕a_{ui}\right)$. Finally, $U_{i}$ stores $\left\{A_{ui},B_{ui},a,A_{ui}⊕a_{ui},\tau_{ui},Y,P,n_{0}\right\}$.

#### 4.1.2. Login and Authentication

If $U_{i}$ wants to access the IoT device $S_{k}$, $U_{i}$ can initiate the following login and authentication request to the gateway as follows:

A1. $U_{i}$ inputs $\left\{ID_{ui}^{*},PW_{ui}^{*},Bio_{ui}^{*}\right\}$ and calculates $\delta_{ui}^{*}=Rep\left(Bio_{ui}^{*},\tau_{ui}\right)$, $RPW_{ui}^{*}=h(PW_{ui}^{*}∥\delta_{ui}^{*}∥a)$, $k_{ui}^{*}=B_{ui}⊕h\left(RPW_{ui}^{*}∥ID_{ui}\right)$, and $A_{ui}^{*}=h(ID_{ui}^{*}∥RPW_{ui}^{*}∥k_{ui}^{*})\;\mathrm{mod}\;n_{0}$. Then, $U_{i}$ verifies whether $A_{i}^{*}\neq A_{i}$ is valid. If it is true, $U_{i}$ selects $r_{ui}$ and computes $a_{ui}^{*}=\left(A_{ui}⊕a_{ui}\right)⊕A_{ui}$, $M_{1}=r_{ui}\cdot Y$, $M_{2}=r_{ui}\cdot P$, $M_{3}=h\left(M_{2}∥M_{1}\right)⊕\left(ID_{ui}^{*}∥a_{ui}^{*}\right)$, $M_{4}=h(M_{1}∥M_{2}∥M_{3})⊕ID_{sk}$, and $M_{5}=h(k_{ui}^{*}∥ID_{i}^{*}∥M_{1}∥M_{2}∥ID_{sk})$. Finally, $U_{i}$ sends $\left\{M_{2},M_{3},M_{4},M_{5}\right\}$ to the cloud server.
A2. After receiving $\left\{M_{2},M_{3},M_{4},M_{5}\right\}$, the cloud server first calculates $M_{1}^{\prime }=y\cdot M_{2}$ and $ID_{ui}^{\prime }∥a_{ui}^{\prime }=M_{3}⊕h\left(M_{2}∥M_{1}^{\prime }\right)$. Then, it retrieves $\left\{T_{rgui},a_{ui}\right\}$ using $ID_{ui}^{\prime }$. If $a_{ui}\neq a_{ui}^{\prime }$, the cloud server terminates the session; otherwise, the cloud server calculates $k_{ui}^{\prime }=h(ID_{ui}^{\prime }∥y∥T_{rg_{ui}})$, $ID_{sk}^{\prime }=M_{4}⊕h(M_{1}∥M_{2}∥M_{3})$, and $M_{5}^{\prime }=h(k_{ui}^{\prime }∥ID_{ui}^{\prime }∥M_{1}^{\prime }∥M_{2}∥ID_{sk}^{\prime })$. If $M_{5}^{\prime }\neq M_{5}$, the cloud server terminates the session; otherwise, it determines the gateway to which $S_{k}$ belongs, selects a random number *r*, and calculates $X_{G_{k}}^{\prime }=h\left(x∥GID_{k}\right)$, $M_{6}=h\left(X_{G_{k}}^{\prime }∥M_{2}\right)⊕r$, $M_{7}=h(M_{6}∥r∥X_{G_{k}}^{\prime })⊕ID_{sk}^{\prime }$, and $M_{8}=h(M_{2}∥M_{6}∥M_{7}∥r∥ID_{sk}^{\prime }∥X_{G_{k}}^{\prime })$. The cloud server then sends $\left\{M_{2},M_{6},M_{7},M_{8}\right\}$ to the gateway.
A3. Upon receiving $\left\{M_{2},M_{6},M_{7},M_{8}\right\}$, the gateway first calculates $r^{\prime }=M_{6}⊕h\left(X_{G_{k}}∥M_{2}\right)$, $ID_{sk}^{\prime \prime }=M_{7}⊕h(M_{6}∥r^{\prime }∥X_{G_{k}})$, and $M_{8}^{\prime }=h(M_{2}∥M_{6}∥M_{7}∥r^{\prime }∥ID_{sk}^{\prime \prime }∥X_{G_{k}})$. Then, it verifies whether $M_{8}^{\prime }=M_{8}$. If it is true, the gateway selects a random number $r_{g}$ and computes $X_{sk}^{\prime }=h\left(x_{G_{k}}∥ID_{sk}^{\prime \prime }\right)$, $M_{9}=h\left(X_{sk}^{\prime }∥M_{2}\right)⊕r_{g}$, and $M_{10}=h(M_{2}∥M_{9}∥r_{g}t∥ID_{sk}^{\prime \prime }∥X_{sk}^{\prime })$. The gateway then sends $\left\{M_{2},M_{9},M_{10}\right\}$ to $S_{k}$.
A4. Upon receiving $\left\{M_{2},M_{9},M_{10}\right\}$, $S_{k}$ calculates $r_{g}^{\prime }=M_{9}⊕h\left(X_{sk}∥M_{2}\right)$ and $M_{10}^{\prime }=h(M_{2}∥M_{9}∥r_{g}^{\prime }∥ID_{sk}∥X_{sk})$. If $M_{10}^{\prime }\neq M_{10}$, $S_{k}$ terminates the session; otherwise, $S_{k}$ selects a random number $r_{sk}$ and then calculates $M=r_{sk}\cdot M_{2}$ and $M_{11}=r_{sk}\cdot P$. Then, $S_{k}$ calculates the session key $SK_{ki}=h(M_{2}∥M_{11}∥M)$ and $M_{12}=h(M_{2}∥M_{11}∥r_{g}^{\prime }∥X_{sk}∥ID_{sk})$. Finally, $S_{k}$ sends $\left\{M_{11},M_{12}\right\}$ to the gateway.
A5. After receiving the response from $S_{k}$, the gateway calculates $M_{12}^{\prime }=h(M_{2}∥M_{11}∥ID_{sk}^{\prime \prime }∥r^{\prime }∥X_{G_{k}})$. If $M_{12}^{\prime }\neq M_{12}$, the gateway terminates the session; otherwise, the gateway calculates $M_{13}=h(M_{11}∥M_{2}∥ID_{sk}^{\prime \prime }∥r^{\prime }∥X_{G_{k}})$ and sends $\left\{M_{11},M_{13}\right\}$ to the cloud server.
A6. After receiving the response from the gateway, the cloud server calculates $M_{13}^{\prime }=h(M_{11}∥M_{2}∥ID_{sk}^{\prime }∥r∥X_{G_{k}}^{\prime })$. If $M_{13}^{\prime }\neq M_{13}$, the cloud server terminates the session; otherwise, it calculates $M_{14}=h(M_{1}^{\prime }∥M_{2}∥ID_{ui}^{\prime }∥ID_{sk}^{\prime }∥k_{ui}^{\prime }∥M_{11})$ and sends $\left\{M_{11},M_{14}\right\}$ to $U_{i}$.
A7. Once $U_{i}$ receives the response from the cloud server, it calculates $M_{14}^{*}=h(M_{1}∥M_{2}∥ID_{i}∥ID_{sk}∥k∥M_{11})$. If $M_{14}^{*}=M_{14}$, $U_{i}$ calculates $SK_{ik}=h(M_{2}∥M_{11}∥r_{i}\cdot M_{11})$ as the session key.

### 4.2. Flaws of Wang et al.’s Protocol

#### 4.2.1. Vulnerability to Insider IoT Device Impersonation Attacks

Consider an attacker as an insider user of the gateway. The attacker first selects a random number $r_{sk}^{*}$ and computes $M^{*}=r_{sk}^{*}\cdot M_{2}$, $M_{11}^{*}=r_{sk}^{*}\cdot P$, and $SK_{ki}^{*}=hM_{2}∥M_{11}^{*}∥M^{*})$. The attacker then calculates $M_{13}^{*\prime }=h(M_{11}^{*}∥M_{2}∥ID_{sk}^{\prime }∥r∥X_{G_{k}}^{\prime })$ and sends $\left\{M_{11}^{*},M_{13}^{*}\right\}$ to the cloud server. Upon receiving $\left\{M_{11}^{*},M_{13}^{*}\right\}$, the cloud server computes $M_{13}^{*\prime }=h(M_{11}^{*}∥M_{2}∥ID_{sk}^{\prime }∥r∥X_{G_{k}}^{\prime })$. Since $M_{13}^{*\prime }=M_{13}^{*}$, the cloud server computes $M_{14}^{*}=h(M_{1}^{\prime }∥M_{2}∥ID_{ui}^{\prime }∥ID_{sk}^{\prime }∥k_{i}^{\prime }∥M_{11}^{*})$ and sends $\left\{M_{11}^{*},M_{14}^{*}\right\}$ to $U_{i}$. Upon receiving the response from the cloud server, $U_{i}$ calculates $M_{14}^{*\prime }=h(M_{1}∥M_{2}∥ID_{ui}∥ID_{sk}∥k∥M_{11}^{*})$. Clearly, $M_{14}^{*}=M_{14}^{*\prime }$, and $U_{i}$ computes $SK_{ik}^{*}=h(M_{2}∥M_{11}^{*}∥r_{ui}\cdot M_{11}^{*})$ as the session key. Similarly, insider users of the cloud server can also initiate impersonation attacks.

#### 4.2.2. Vulnerability to ESL Attacks

If $r_{ui}$ is compromised, the attacker can combine the channel messages $\left\{M_{2},M_{6},M_{7},M_{8}\right\}$ and $\left\{M_{11},M_{14}\right\}$ to calculate $SK_{ik}=h(M_{2}∥M_{11}∥r_{i}\cdot M_{11})$. Similarly, if $r_{sk}$ is leaked, the attacker can combine channel messages $\left\{M_{2},M_{6},M_{7},M_{8}\right\}$ and $\left\{M_{11},M_{14}\right\}$ to compute $M=r_{sk}\cdot M_{2}$, $M_{11}=r_{sk}\cdot P$, and $SK_{ki}=h(M_{2}∥M_{11}∥M)$. Thus, the scheme is vulnerable to ESL attacks.

## 5. Proposed Protocol

The proposed protocol has four phases: system initialization, registration, login and authentication, and user password and biometric update.

### 5.1. System Initialization

The implementation of the system is carried out by RC $S_{j}$ online. Firstly, $S_{j}$ chooses an elliptic curve E(a,b) over $Z_{q}^{*}=\left\{0,1,\cdots ,q-1\right\}$ with a base point *P*. In addition, a medium number $d=10^{8}$ is chosen. Second, RC opts for its identifier $ID_{sj}\in Z_{q}^{*}$ and its long-term private key $s_{sj}\in Z_{q}^{*}$ and computes $PK_{sj}=s_{sj}\cdot P$ as its public key. Then, RC chooses two one-way hash functions, $h\left(\cdot \right):{\left\{0,1\right\}}^{*}\to {\left\{0,1\right\}}^{256}$ and $h1\left(\cdot \right):{\left\{0,1\right\}}^{*}\to {\left\{0,1\right\}}^{64}$, for generating hashes and one-time credential identifiers, respectively. Finally, RC keeps $s_{sj}$ secure and issues the public parameters of the system $\left\{E\left(a,b\right),q,P,ID_{sj},d,h\left(\cdot \right),h1\left(\cdot \right)\right\}$.

### 5.2. Registration

#### 5.2.1. IoT Device Registration

IoT device $S_{k}$ selects an RC $S_{j}$ to complete registration and obtain authentication credentials before the entry of the system. The registration process is as follows:

*R1:*  $S_{k}$ selects its identifier $ID_{sk}\in Z_{q}^{*}$ and a random number $d_{sk}\in Z_{q}^{*}$ and then computes $D_{sk}=H\left(ID_{sk}∥d_{sk}\right)\cdot P$. Third, $S_{k}$ sends the registration requisition request message, {$ID_{sk},D_{sk}$}, to $S_{j}$ through secure channels.
*R2:*  In response, $S_{j}$ checks if $ID_{sk}$ has been registered. If so, $S_{k}$ is required for a new request. Otherwise, RC opts for a random number $x_{sk}\in Z_{q}^{*}$ as the partial private key of $S_{k}$ and computes $PK_{sk}=D_{sk}+x_{sk}\cdot P$ as the public key of $S_{k}$. After that, RC publishes $\left\{ID_{sk},PK_{sk}\right\}$ and replies $\left\{x_{sk},PK_{sk}\right\}$ to $S_{k}$ through secure channels. Lastly, RC computes $w_{sk}=h\left(s_{sj}\cdot PK_{sk}∥ID_{sk}\right)$ and stores $\left\{w_{sk}\right\}$ secretly.
*R3:*  After receiving $\left\{x_{sk},PK_{sk}\right\}$, $S_{k}$ computes $s_{sk}=\left(h\left(ID_{sk}∥d_{sk}\right)+x_{sk}\right)\;\mathrm{mod}\;q$ as its private key. Then, $S_{k}$ checks whether $s_{sk}\cdot P?=PK_{sk}$. If it holds, $S_{k}$ computes $w_{sj}=h\left(s_{sk}\cdot PK_{sj}∥ID_{sk}\right)$ and stores $\left\{ID_{sk},s_{sk},PK_{sk},w_{sj}\right\}$.

**Remark** **1.**
$w_{sj}=h\left(s_{sk}\cdot PK_{sj}∥ID_{sk}\right)=h\left(s_{sk}s_{sj}\cdot P∥ID_{sk}\right)=h\left(s_{sj}\cdot PK_{sk}∥ID_{sk}\right)=w_{sk}$

#### 5.2.2. User Registration and Revocation

**Figure 3:**
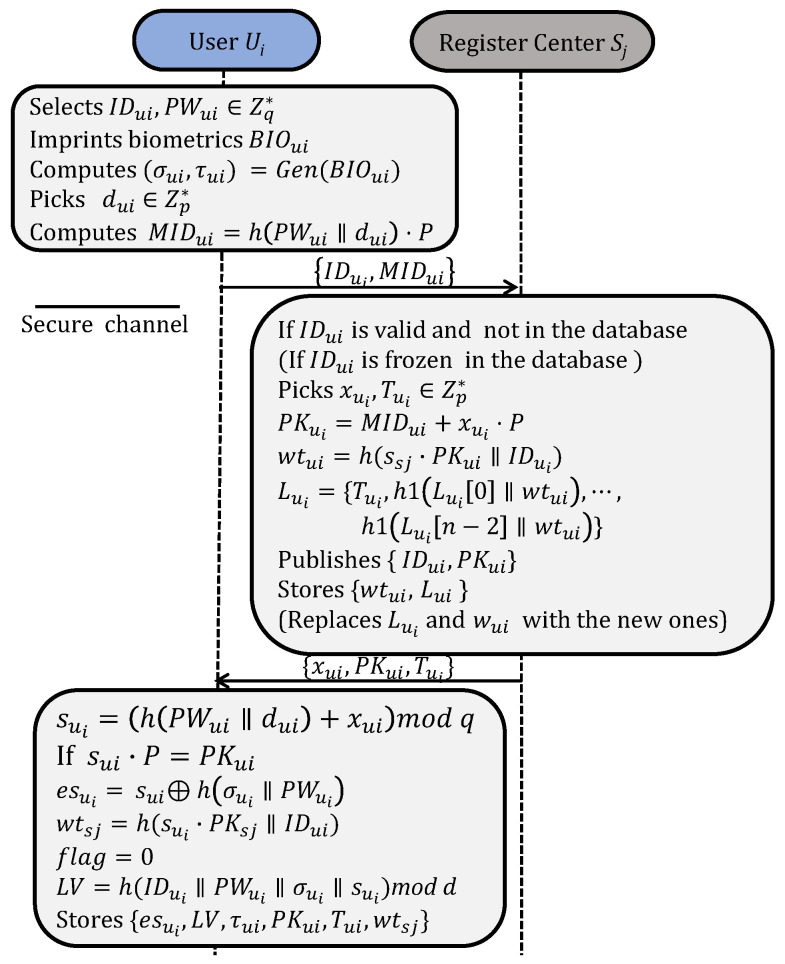
User registration and revocation.

Users can dynamically join different systems through the registration processes shown in Figure 3. The *fuzzy verifier* $LV=h(ID_{ui}∥PW_{I}D_{ui}∥\sigma_{I}D_{ui}∥s_{I}D_{ui})\;\mathrm{mod}\;d$ [2,44] is used to eliminate offline password guessing attacks. $T_{ui}$ is a temporary one-time credential assigned to the user $U_{i}$ and is marked with flag=0 to indicate that $T_{ui}$ has not been used. A corresponding untraceable list $L_{ui}=\left\{T_{ui},h1\left(L_{ui}\left[0\right]∥wt_{ui}\right),\cdots ,h1\left(L_{ui}\left[n-2\right]∥wt_{ui}\right)\right\}$ is generated and stored in RC, where *n* represents the number of times a user is permitted to re-initiate the authentication. After registration, $U_{i}$ stores $\left\{es_{ui},LV,\tau_{ui},PK_{ui},T_{ui},wt_{sj}\right\}$. $S_{j}$ publishes $\left\{ID_{ui},PK_{ui}\right\}$ and stores $\left\{wt_{ui},L_{ui}\right\}$ secretly. A user account can be updated with the same process if it is frozen.

### 5.3. Login and Authentication

**Figure 4:**
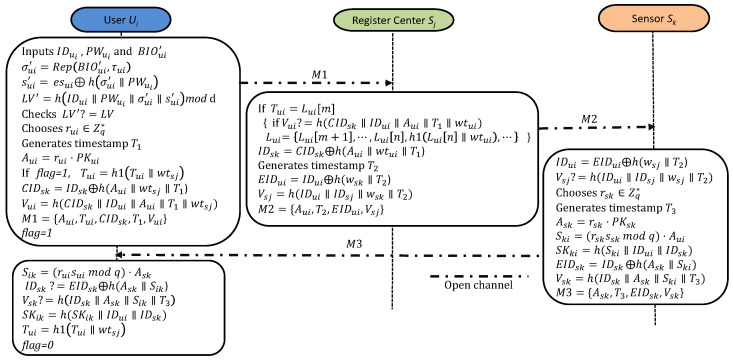
Login and authentication.

This phase between the user $U_{i}$, the RC $S_{j}$, and the sensor $S_{k}$ is shown in Figure 4.

*A1:*  To begin, $U_{i}$ inputs $ID_{ui}$, $PW_{ui}$, and $BIO_{ui}^{\prime }$. Then, $U_{i}$ calculates $\sigma_{ui}^{\prime }=\mathrm{Rep}\left(BIO_{ui}^{\prime },\tau_{ui}\right)$ and $s_{ui}^{\prime }=es_{ui}⊕h\left(\sigma_{ui}^{\prime }∥PW_{ui}\right)$ and obtains a verifier $LV^{\prime }=h(ID_{ui}∥PW_{ui}∥\sigma_{ui}^{\prime }∥s_{ui}^{\prime })\;\mathrm{mod}\;n$. Finally, $U_{i}$ verifies if $LV^{\prime }?=LV$. If this condition is not met, the log-in process will end.
*A2:*  $U_{i}$ chooses a random $r_{ui}\in Z_{q}^{*}$ and generates the current timestamp $T_{1}$. Afterwards, $U_{i}$ calculates $A_{ui}=r_{ui}\cdot PK_{ui}$. If flag=1, it indicates that $T_{ui}$ has been used. In this case, $U_{i}$ updates $T_{ui}$ to $T_{ui}=h1\left(T_{ui}∥wt_{sj}\right)$. Third, $U_{i}$ encodes the identity of the desired sensor $S_{k}$ into dynamic identity as $CID_{sk}=ID_{sk}⊕h(A_{ui}∥wt_{sj}∥T_{1})$. Finally, $U_{i}$ generates a verifier $V_{ui}=h(CID_{sk}∥ID_{ui}∥A_{ui}∥T_{1}∥wt_{sj})$ and sends the request message $M1=\{A_{ui},T_{ui},CID_{sk},T_{1},V_{ui}\}$ to $S_{j}$.
*A3:*  Upon receiving M1, $S_{j}$ verifies its freshness with the timestamp $T_{1}$. Next, $S_{j}$ verifies $U_{i}$ using temporary credentials $T_{ui}$. If $T_{ui}=L_{ui}\left[m\right]\left(m\leq n\right)$ and $V_{ui}=h(CID_{sk}∥ID_{ui}∥A_{ui}∥T_{1}∥wt_{ui})$, then $S_{j}$ confirms that the sender is $U_{i}$ and updates the untraceable list for $T_{ui}$ as follows: $L_{ui}=\left\{L_{ui}\left[m+1\right],\cdots ,L_{ui}\left[n\right],h1\left(L_{ui}\left[n\right]∥wt_{ui}\right),\cdots \right\}$. Otherwise, $S_{j}$ will reject the session.
*A4:*  $S_{j}$ generates the time stamp $T_{2}$ and encodes the identity of $U_{i}$ as $EID_{ui}=ID_{ui}⊕h\left(w_{sk}∥T_{2}\right)$. Finally, $S_{j}$ generates a verifier $V_{sj}=h(ID_{ui}∥ID_{sj}∥w_{sk}∥T_{2})$ and sends the message $M2=\{A_{ui},T_{2},EID_{ui},V_{sj}\}$ to the IoT device $S_{k}$.
*A5:*  Upon receiving *M2*, $S_{k}$ checks its freshness with the timestamp $T_{2}$. Following that, $S_{k}$ decodes $ID_{ui}=EID_{ui}⊕h\left(w_{sj}∥T_{2}\right)$. Finally, $S_{k}$ verifies the authenticity of $S_{j}$ by checking whether $V_{sj}?=h(ID_{ui}∥ID_{sj}∥w_{sj}∥T_{2})$ and ensures the integrity of the message received. If not, $S_{j}$ will terminate the verification immediately.
*A6:*  Firstly, $S_{k}$ selects a random $r_{sk}\in Z_{q}^{*}$ and generates the current timestamp $T_{3}$. After that, $S_{k}$ calculates $A_{sk}=r_{sk}\cdot PK_{sk}$, $S_{ki}=\left(r_{sk}s_{sk}\;\mathrm{mod}\;q\right)\cdot A_{ui}$, and obtains the session key shared with $U_{i}$ as $SK_{ki}=h(S_{ki}∥ID_{ui}∥ID_{sk})$. Then, $S_{k}$ encodes $EID_{sk}=ID_{sk}⊕h\left(A_{sk}∥S_{ki}\right)$. Third, $S_{j}$ generates its verifier as $V_{sk}=h(ID_{sk}∥A_{sk}∥S_{ki}∥T_{3})$. Finally, $S_{j}$ transmits the response message $M3=\{A_{sk},T_{3},EID_{sk},V_{sk}\}$ to $U_{i}$.
*A7:*  Upon receiving *M3*, $U_{i}$ first checks its freshness with the timestamp $T_{3}$. After that, $U_{i}$ calculates $S_{ik}=\left(r_{ui}s_{ui}\;\mathrm{mod}\;q\right)\cdot A_{sk}$. Third, $U_{i}$ verifies whether $ID_{sk}?=EID_{sk}⊕h\left(A_{sk}∥S_{ik}\right)$ and $V_{sk}?=h(ID_{sk}∥T_{ui}∥A_{sk}∥S_{ik}∥T_{3})$. If not, $U_{i}$ will terminate the session. Finally, $U_{i}$ obtains the session key shared with $S_{k}$ as $SK_{ik}=h(S_{ik}∥ID_{ui}∥ID_{sk})$ and updates $T_{ui}$ as $T_{ui}=h1\left(T_{ui}∥wt_{sj}\right)$ with flag=0.

### 5.4. User Password and Biometric Update

A registered user $U_{i}$ can renew her password and biometric information following the steps outlined below.

$U_{i}$ first inputs $ID_{ui},\;PW_{ui}$, and $BIO_{ui}^{\prime }$ and then computes $\sigma_{ui}^{\prime }=\mathrm{Rep}\left(BIO_{ui}^{\prime },\tau_{ui}\right)$, $s_{ui}^{\prime }=es_{ui}⊕h\left(\sigma_{ui}^{\prime }∥PW_{I}D_{ui}\right)$, and $LV^{\prime }=h(h(ID_{ui}∥PW_{I}D_{ui}∥\sigma_{ui}^{\prime }∥s_{ui}^{\prime })\;\mathrm{mod}\;n)$. Third, $U_{i}$ checks if $LV^{\prime }?=LV$. If so, $U_{i}$ inputs $PW_{ui}^{new}$ and $BIO_{ui}^{new}$ and computes $\left(\sigma_{ui}^{new},\tau_{ui}\right)=\mathrm{Gen}t\left(BIO_{ui}^{new}\right)$, $es_{ui}^{new}=s_{ui}⊕h\left(\sigma_{ui}^{new}∥PW_{ui}^{new}\right)$, and $LV^{new}=h(ID_{ui}∥RPW_{ui}^{new}∥\sigma_{ui}^{new}∥s_{I}D_{ui})\;\mathrm{mod}\;n$. Finally, $U_{i}$ replaces $\left\{es_{ui},LV\right\}$ with $\left\{es_{ui}^{new},LV^{new}\right\}$.

## 6. Formal Security Analysis

This section provides the formal proof of the proposed protocol in the IoT-3eCK security model.

### 6.1. Relevant Definitions

**Participants:** The proposed protocol involves three participants: $U_{i}$, $S_{j}$, and $S_{k}$. Each participant has several instances denoted by $I_{U}^{i}$, $I_{S}^{j}$, and $I_{S}^{k}$(i,j,k∈Z), respectively. Any instance can be referred to as $I\in I_{U}^{i}\cup I_{S}^{j}\cup I_{S}^{k}$. A session identifier, Sid, is created by concatenating all messages sent and received for the current session. An instance can exist in three states. If the most recent expected protocol message is received, the instance enters the state accept. If an incorrect message is received, the instance transitions to the reject state. An instance will become ⊥ if the input is not responded to.

**Partners:** Instances *I* and $I^{\prime }$ are considered partners only if all three conditions are met: (i) Both instances are currently in the accept state. (ii) They have mutually authenticated and shared the same Sid. (iii) They are partners with each other.

**Freshness:** An instance is considered *fresh* if A fails to obtain the session key $SK_{ki}$ (or $SK_{ik}$) between $U_{i}$ and $S_{k}$ by making SKReveal(I), ESReveal (I), or Corrupt(I) queries before performing a Expire(I) query.

**Adversary:** Under the IoT-3eCK adversary model, A can interact with $U_{i}$, $S_{j}$, and $S_{k}$ through the following queries:

(1) Execute(): A can observe all messages transmitted between $U_{i}$, $S_{j}$, and $S_{k}$.
(2) Send(I,m): A can send a message *m* to *I* and receive a response.
(3) ESReveal(I): This query reveals the ephemeral secret key of *I* to A.
(4) SKReveal(I): A can obtain the session key between *I* and its partner.
(5) UCorrupt(w): A can obtain the security credentials held by $U_{i}$.
  - w=0: A acquires $PW_{ui}$ and $BIO_{ui}$.
  - w=1: A acquires $PW_{ui}$ and $\left\{es_{ui},LV,\tau_{ui},T_{ui},wt_{sj}\right\}$.
  - w=2: A acquires $BIO_{ui}$ and $\left\{es_{ui},LV,\tau_{ui},T_{ui},wt_{sj}\right\}$.
(6) RCCorrupt(): A can retrieve all the security credentials held by $S_{j}$.
(7) SCorrupt(): A can retrieve all the security credentials held by $S_{k}$.
(8) Test(I): This query is used to simulate the semantic security of the session key.
  - If the session key is not established or *I* is not *fresh*, ⊥ will be returned.
  - Otherwise, a coin b∈{0,1} is flipped.
    − If b=1, *I* returns the session key to A.
    − If b=0, *I* returns a random string of the same length as the session key.
(9) h(M): The query provides A with a randomly generated number that serves as the hash output of *M*.
(10) Expire(I): Once this query is executed, the session key held by *I* is deleted.

### 6.2. Provable Security Analysis

Before the security analysis, we first define the complexity assumptions. We then provide a formal proof to evaluate the security of the session key $SK_{ik}$ (or $SK_{ki}$) under the ROM model.

Let E(a,b) denote a non-singular elliptic curve over a finite field $F_{q}$, *G* be an additive group of order *q* of E(a,b), and *P* be the base point of the group.

**Definition** **1.** Elliptic Curve Discrete Logarithm (ECDL) Hardness Assumption: Given <P,xP∈G>, finding $x\in Z_{q}^{*}$ is computationally hard, and the probability that A is able to resolve the problem, ${\mathrm{Adv}}_{\mathrm{ECDL}}\left(A\right)$, is negligible for sufficiently small ϵ,

(1) $$Adv_{\mathrm{ECDL}}\left(A\right)=Pr\left[A\left(P,xP\right)=x:x\in Z_{q}^{*}\right]<\epsilon$$

**Definition** **2.** *Computational Diffie–Hellman (CDH) Hardness Assumption: Given <xP,yP∈G>, figuring out xyP∈G is computationally hard, and the probability of A resolving the problem is negligible for sufficiently small ϵ,*

(2) $$Adv_{\mathrm{CDH}}\left(A\right)=Pr\left[A\left(xP,yP\right)=xyP:x,y\in Z_{q}^{*}\right]<\epsilon$$

**Definition** **3.** *The security of $SK_{ik}$ (or $SK_{ki}$) is modeled by the game $Game_{\mathrm{UA}}\left(I,A\right)$. In this game, A is allowed to perform one Test(I) query, where I is both accept and fresh, and I returns a single bit $b^{\prime }$. A can issue many other queries to I.*

Let Pr[Succ(A)] denote the probability that A successfully wins the game $Game_{\mathrm{UA}}\left(I,A\right)$. The advantage of A in breaking the *semantic security* of $SK_{ik}$ (or $SSK_{ki}$) is defined as

(3) $$Adv_{\mathrm{UA}}\left(A\right)=|2Pr\left[b^{\prime }=b\right]-1\left|=\right|2PrSucc\left(A\right)-1|$$

**Definition** **4.** *In the context of the adversarial model eCK, a user authentication scheme is semantically secure if $Adv_{\mathrm{UA}}\left(A\right)$ is negligible for any adversary.*

**Theorem** **1.** *Let A be an adversary that can break the semantic security of $SK_{ik}$ (or $SSK_{ki}$) by creating up to $q_{e}$Execute() queries and $q_{h}$h() and $q_{s}$Send() queries, all within polynomial time t. Let the random numbers and hash outputs be l-bit values, the password dictionary space be N, following Zipf’s law as in [17], and the biometric secret key have a length of lb bits with a false-positive probability of $\varepsilon_{fp}$. Then, the advantage of A is that*

(4) $$\begin{array}{cc} Adv_{\mathrm{UA}}\left(A\right)\leq & \frac{\left(q_{h}^{2}+3q_{h}+3q_{s}\right)}{2^{l}}+\frac{{\left(q_{s}+q_{e}\right)}^{2}}{2(q-1)}+(2\max\left\{\frac{q_{s}}{2^{lb}},\frac{q_{s}}{2^{lb}}+q_{s}\varepsilon_{b},C^{\prime }q_{s}^{s^{\prime }}\right\}+\frac{3q_{s}}{2^{l}}\\ \; & +\frac{3q_{s}}{q-1})\max\left\{Adv_{\mathrm{ECDL}}\left(A\right),\mathrm{Ad}v_{\mathrm{CDH}}\left(A\right)\right\} \end{array}$$

*where $C^{\prime }$ and $s^{\prime }$ are Zipf’s parameters.*

**Proof.**  The *semantic security* of $SSK_{ik}$ (or $SSK_{ki}$) is defined by a series of games ${\mathrm{Game}}_{I}\left(i=0,1,\cdots ,5\right)$. In these games, $S_{i}$ represents the value of *b* in the Test(I) query correctly predicted by A, and $Pr[S_{i}]$ denotes the probability that the event $S_{i}$ will occur.

$Game_{0}$: The game begins by simulating a real attack in ROM mode. Thus, we have the following:

(5) $$Adv_{UA}\left(A\right)=|2Pr\left[S_{0}\right]-1|$$

$Game_{1}$: In this game, A can be the Execute() query to obtain M1, M2, and M3 exchanged between $(U_{i}$, $S_{j}$, and $S_{k}$. Then, A can use SKReveal(I) and Test(I) to verify whether the calculated key $SSK_{ki}$ (or $SK_{ik}$) is genuine or not. Since $SK_{ki}$ (or $SK_{ik}$) cannot be determined directly from these messages, there is no distinguishable difference between $Game_{0}$ and $Game_{1}$. Therefore,

(6) $$Pr\left[S_{1}\right]=Pr\left[S_{0}\right]$$

Three lists are used to store related results: $L_{h}$ for the inputs and outputs of h() queries, $L_{h}^{A}$ for the answers to h() queries asked by A, and $L_{E}$ for the inputs and outputs of Execute() queries.

${Game}_{2}$: The game ends when message transcripts and hash queries collide. The components of messages M1, M2, and M3 are evenly distributed. Based on the birthday paradox, we obtain

(7) $$|Pr\left[S_{2}\right]-Pr\left[S_{1}\right]|\leq \frac{{\left(q_{s}+q_{e}\right)}^{2}}{2(q-1)}+\frac{q_{h}^{2}}{2^{l+1}}$$

$Game_{3}$: In this game, A attempts to forge the messages M1, M2, and M3 without access to the oracle. The process is as follows:

- Constructing M1: A needs to issue h() queries to successfully construct M1. Thus, the following values must be in $L_{h}^{A}$: ($A_{ui}∥B_{ui},*),(ID_{sk}∥ID_{ui}∥B_{ui}∥T_{ui}∥*,V_{ui})$. The probability of success is $\frac{\left(q_{h}+q_{s}\right)}{2^{l}}$.
- Constructing M2: Similarly, we have $\left(*∥T_{sj},*\right)$, $(ID_{ui}∥ID_{sj}∥*∥T_{sj},V_{sj})\in L_{h}^{A}$, and the probability is $\frac{\left(q_{h}+q_{s}\right)}{2^{l}}$.
- Constructing M3: Again, we have $\left(A_{sk}∥*,*\right)$, $(ID_{sk}∥ID_{ui}∥*∥T_{sk}∥V_{sk})\in L_{h}^{A}$, and the probability is $\frac{\left(q_{h}+q_{s}\right)}{2^{l}}$.

Thus, $Game_{3}$ is indistinguishable from game $Game_{2}$ unless A successfully constructs M1, M2, and M3. Thus,

(8) $$|Pr\left[S_{3}\right]-\mathrm{Pr}\left[S_{2}\right]|\leq \frac{3(q_{h}+q_{s})}{2^{l}}$$

$Game_{4}$: This game terminates if A is able to retrieve $SK_{ki}$ (or $SK_{ik}$). If A manages to compute the session key, this implies that it has solved the ECDL or ECD problems. Furthermore, the tuple $\left(r_{ui}s_{ui}r_{sk}s_{sk}\cdot P∥ID_{i}∥ID_{j}\right)$ must be stored in $L_{h}^{A}$. The following cases may occur for A to compute $SK_{ki}$ or $SK_{ik}$:

(1) A issues UCorrupt(w) and SCorrupt().
  - w=0: A acquires $PW_{ui}$ and $BIO_{ui}$, and then A guesses $s_{ui}$ in Send() queries with a success probability of $\frac{q_{s}}{2^{l}}$.
  - w=1: A acquires $PW_{ui}$ and $\left\{es_{ui},LV,\tau_{ui},T_{ui},wt_{sj}\right\}$, and then A attempts to obtain $BIO_{ui}$ using two methods. A can guess $\delta_{ui}$ in Send() queries with lb bits with probability $\frac{q_{s}}{2^{lb}}$, or replace $BIO_{ui}$ with collected biometric data with a probability of $q_{s}\cdot \varepsilon_{b}$, where $\varepsilon_{b}$ is the probability of similarity of the biometric data. So, the probability is $\frac{q_{s}}{2^{lb}}+q_{s}\cdot \varepsilon_{b}$.
  - w=2: A acquires $BIO_{ui}$ and $\left\{es_{ui},LV,\tau_{ui},T_{ui},wt_{sj}\right\}$, and then A attempts to guess $PW_{ui}$ in $q_{s}$ queries. According to Zipf’s law, the probability of password distribution is $C^{\prime }q_{s}^{s^{\prime }}$, where $C^{\prime }$ and $s^{\prime }$ are Zipf’s parameters.
  Next, A issues an SCorrupt() to obtain $s_{sk}$ of $S_{k}$, and then A attempts to guess $r_{sk}$ using $q_{s}$ queries with the probability of $\frac{q_{s}}{q-1}$.
(2) A issues UCorrupt(w) and $ESReveal(S_{k})$.
  Firstly, A issues a UCorrupt(w) query as summarized above. Next, A issues an $ESReveal(S_{k})$ query to obtain the ephemeral secret $r_{sk}$ of $S_{k}$. Then, A attempts to guess $s_{sk}$ within $q_{s}$ attempts, with a probability of $\frac{q_{s}}{2^{l}}$.
(3) A issues $ESReveal(U_{i})$ and SCorrupt().
  Firstly, A obtains $r_{ui}$ and $s_{sk}$. Then, A attempts to guess $s_{sk}$ and $r_{sk}$.
(4) A issues $ESReveal(U_{i})$ and $ESReveal(S_{k})$.
  Firstly, A obtains $r_{ui}$ and $r_{sk}$. Then, A tries to guess $s_{sk}$ and $s_{sk}$.

In all the above cases, A cannot figure out $SK_{ik}$ (or $SK_{ik}$) unless it has solved the problem of ECDL or ECD. Hence,

(9) $$\begin{array}{c} |Pr\left[S_{4}\right]-Pr\left[S_{3}\right]|\leq (2\max\left\{\frac{q_{s}}{2^{l}},\frac{q_{s}}{2^{lb}}+q_{s}\varepsilon_{b},C^{\prime }q_{s}^{s^{\prime }}\right\}+\\ \frac{3q_{s}}{\left(q-1\right)}+\frac{3q_{s}}{2^{l}})\max\left\{Adv_{\mathrm{ECDL}}\left(A\right),Adv_{\mathrm{CDH}}\left(A\right)\right\} \end{array}$$

$Game_{5}$: This game emulates $Game_{4}$, but the Test() query will terminate if A issues an $h(S_{ik}∥ID_{ui}∥ID_{sk})$ query. The probability of A acquiring ${SK}_{ik}$ is at most $q_{h}^{2}/2^{l+1}$. Therefore,

(10) $$|Pr\left[S_{5}\right]-Pr\left[S_{4}\right]|\leq q_{h}^{2}/2^{l+1}$$

Without proper input to the h() query, A cannot differentiate the actual session key from a random one. Therefore,

(11) $$Pr[S_{5}]=1/2$$

Given all the probabilities and conditions above, Theorem 1 holds. □

## 7. Automatic Verification Using ProVerif

ProVerif is a powerful tool for the formal verification of cryptographic protocols. Written in Prolog, it uses Horn clauses and PI algorithms to perform its tasks. ProVerif is capable of precisely modeling a variety of cryptographic primitives, including shared-key cryptography, public-key cryptography, hash functions, and Diffie–Hellman key-exchange protocols, along with supporting rewriting rules and equations. The tool can demonstrate several important security properties, such as reachability properties, correspondence assertions, and observational equivalence.

The proposed scheme has been verified formally using ProVerif. Table 3 illustrates the processes for $U_{i}$, as outlined in Section 4. In this setup, schij represents a private channel that facilitates communication between $U_{i}$ and $S_{j}$ during the registration phase, while chij and chki are public channels, with $U_{i}$ and $S_{j}$ communicating over chij and $U_{i}$ and $S_{k}$ communicating over chki during login and authentication. The three participants execute their respective processes, and the scheme is simulated to run in parallel as ((!User)|(!Sk)|(!RC)).

As shown in Table 4, queries and results confirm that A is unable to break the authentication process or determine session keys, ephemeral keys, and long-term keys. The proposed scheme ensures high levels of anonymity, consistency, and mutual authentication.

## 8. Analysis of Security Features

### 8.1. User Anonymity and Untracibility

During authentication, each $T_{ui}$ is used only once, regardless of whether authentication is successful, and the values of $T_{ui}$ and other authentication parameters are unique for each session. Furthermore, due to the secrecy of $w_{sj}$ and $w_{ui}$, even insider users of RC cannot determine $T_{ui}$. As a result, the scheme ensures anonymity and untraceability.

### 8.2. Perfect Forward Security

Even if long-term secrets $s_{ui}$ and $s_{sk}$ are compromised, A is still unable to deduce the session key $SK_{ik}$, for $SK_{ik}=h(S_{ik}∥ID_{ui}∥ID_{sk})$, where $S_{ik}=\left(r_{ui}s_{ui}\;\mathrm{mod}\;q\right)\cdot A_{sk}$ and $A_{sk}=r_{sk}\cdot PK_{sk}$. This is due to the fact that the ephemeral secrets $r_{ui}$ and $r_{sk}$ are unknown to A. Also, even if A is aware of $s_{ui}$ and $s_{sk}$, he/she cannot deduce $SK_{ki}$.

### 8.3. Key Privacy

During registration, the system does not store or expose password-related data, nor are any password authentication tables maintained. The user information $\left\{ID_{ui},PK_{ui},T_{ui},wt_{ui}\right\}$ maintained by RC does not consist of the user’s password and its variants. The long-term keys of $U_{i}$ and $S_{k}$ are neither generated by the RC nor known to it. Furthermore, the component of session keys $S_{ik}=\left(r_{ui}s_{ui}\;\mathrm{mod}\;q\right)\cdot A_{sk}$ and $S_{ki}=\left(r_{sk}s_{sk}\;\mathrm{mod}\;q\right)\cdot A_{ui}$ does not involve RC. Due to the hardness of the ECDL and ECD problems, A cannot figure out $s_{ui}$ and $s_{sk}$ even if $S_{ik}$ or $S_{ki}$ are exposed. In a nutshell, no insider user of RC can break the security of the proposal.

### 8.4. Ephemeral Secret Leakage Attack Resistance

Resistance to ESL attacks comprises the fact that, even if A captures the ephemeral keys, $r_{ui}$ and $r_{sk}$, he/she is unable to obtain the session key $SK_{ik}$ or $SSK_{ki}$. For $SK_{ik}=h(S_{ik}∥D_{ui}∥ID_{sk})$, where $S_{ik}=\left(r_{ui}s_{ui}\;\mathrm{mod}\;q\right)\cdot A_{sk}$ and $A_{sk}=r_{sk}\cdot PK_{sk}$, even if $r_{ui}$ and $r_{sk}$ are compromised, A cannot deduce $SK_{ik}$ because the long-term keys $s_{ui}$ and $s_{sk}$ are unknown to him. Also, even if A is aware of $r_{ui}$ and $r_{sk}$, she cannot deduce $SSK_{ki}$.

### 8.5. IoT Node Capture Attack Resistance

Sensors may be positioned in unattended environments and physically captured by A. The credentials $\left\{ID_{sk},s_{sk},PK_{sk},ID_{sj},w_{sj}\right\}$ can then be easily retrieved. In the protocol, different sensors have different credentials. Therefore, it only leads to the disclosure of session keys between $S_{k}$ and user $U_{i}$, but not between the uncorrupted sensor $S_{k}^{\prime }$ and user $U_{i}^{\prime }$. In other words, the proposed scheme is resistant to IoT node capture attacks and limits the impact of such compromises to the specific nodes affected.

### 8.6. Timely Typo Detection

The parameter LV stored on the user’s device enables immediate validation of user inputs. If the user inputs an incorrect ID or password, the computed value $LV^{\prime }$ will differ from the stored value LV. As a result, the log-in request will be rejected, ensuring prompt detection and mitigation of typographical errors during authentication.

## 9. Performance Comparison

### 9.1. Security Feature Comparison

As shown in Table 5, the comparison of security features indicates that the proposal offers better security and functionality than the related scheme [17,19,20,21,22,25,26]. Compared to existing schemes, the recommended approach effectively provides anonymity, untraceability, PFS, key privacy, and resistance to insider impersonation, ESL, man-in-the-middle, and other attacks.

### 9.2. Communication Cost

Assume that the lengths of the random number (R), hash output (H), identity (ID), ECC point (G), and timestamp (TS) are 128, 256, 256, 384, and 32 bits, respectively. In the proposed scheme, three messages M1, M2, and M3 are transmitted during the login and authentication phase, where $M1=\{A_{ui},TID_{ui},CID_{sk},T_{1},V_{ui}\}$, $M2=\{A_{ui},T_{2},EID_{ui},V_{sj}\}$, and $M3=\{A_{sk},T_{3},EID_{sk},V_{sk}\}$. The lengths of M1, M2, and M3 are 384+128+128+32+256=928 bits, 384+32+128+256=800 bits, and 384+32+128+256=800 bits. The total communication cost is {928+800+800=2528} bits. The communication overheads of [17,19,20,21,22,25,26] are 3584, 4384, 3296, 3872, 3680, 3456, and 3392 bits, respectively. Table 6 shows that the proposed scheme reduces communication costs from 23% to 42%.

### 9.3. Computation Cost

To compare the computational overhead of different schemes, the run times of other cryptographic primitives are tested. A laptop with Intel Core i5-8250U 1.60 GHZ + 16 GB RAM (Intel Corporation, Santa Clara, CA, USA) and Windows 11 serves as the server. A Raspberry Pi 3 Model B+ (Raspberry Pi Ltd., Cambridge, UK) board with ARM Cortex-A53 1.4 GHz + 1 GB RAM (ARM Ltd., Cambridge, UK) serves as the sensor node and user. When *p* = $2^{192}$, the elliptic curve is Curve25519, and the point length is 384 bits; the average running time is shown in Table 7. Table 8 illustrates that our scheme reduces computation costs by comparing 22% to 56% with the related scheme. In particular, the computational overhead of the intermediate server is reduced by more than 95%. These results highlight significant optimization of resource consumption in the proposed scheme, particularly in resource-constrained IoT environments and scenarios where the intermediate server is connected to many nodes.

## 10. Conclusions

In IoT systems such as smart homes and smart healthcare, IoT user authentication protocols enable secure authentication and session key negotiation between users and IoT devices through an intermediate server, thereby ensuring that users can securely access sensor data directly or control IoT devices remotely. However, the existing IoT user authentication schemes typically assume that the server is fully trusted, ignoring the risk of potential attacks from inside entities. For example, an insider user may launch an insider impersonation attack and steal the session key negotiated between the user and the end device. In addition, most of the existing protocols lack key security properties, such as effective defense against ESL attacks and offline password guessing attacks, as well as perfect forward security. Furthermore, as the scale of IoT devices grows dramatically, intermediate servers need to manage a large number of user and device connections, making the performance of authentication schemes highly dependent on the computational power of the servers.

The design of security protocols is based on the security model, and the existing security model for IoT user authentication still has limitations, making it difficult to comprehensively deal with insider attacks and temporary random number leakage. To enhance the security of IoT user authentication protocols, this paper proposes a new security model for IoT user authentication, IoT-3eCK, which assumes that the intermediate server is semi-trusted and enhances the attacker model to improve the security requirements of IoT user authentication protocols. Based on this security model, this paper designs an enhanced and efficient IoT user authentication protocol. In the registration phase, the protocol ensures that long-term secrets, user passwords, and biometric data of users and end devices are not deduced by insider users in the registration center, effectively preventing internal attacks. In the authentication phase, the protocol combines a dynamic pseudo-identity anonymization mechanism and an ECC key-exchange mechanism to ensure that the protocol satisfies additional security properties. The performance analysis shows that, compared to the existing schemes, the protocol reduces the communication cost by more than 22% and the computational overhead by more than 23%, especially on the intermediate server side by more than 95%, which significantly improves system efficiency. In addition, the security of the new protocol is rigorously proved using a randomized predicate machine model and verified using an automated verification tool.

## Figures and Tables

**Table 1:** Summary of relevant ECC-based user AKA protocols.

Scheme	Year	Shortcomings
Jiang et al. [9]	2016	Prone to offline password guessing, ESL, and insider sensor impersonation attacks; lacking key privacy, FPS, and timely typo detection.
Wu et al. [10]	2017	Prone to offline password guessing, ESL, and insider sensor impersonation attacks; lacking untraceability, key privacy, and FPS.
Challa et al. [12]	2017	Prone to offline password guessing, impersonation, and insider IoT device impersonation attacks; lacking key privacy, anonymity, and untraceability; has high computation.
Li et al. [13]	2018	Prone to offline password guessing, replay, ESL, insider sensor impersonation, and impersonation attacks; lacking key privacy.
Li et al. [14]	2018	Prone to offline password guessing, insider sensor impersonation, and ESL attacks; lacking user untraceability and PFS.
Shuai et al. [15]	2019	Prone to offline password guessing, insider IoT device impersonation, and ESL attacks; lacking user untraceability, key privacy, and PFS.
Lu et al. [28]	2019	Based on the sensor ID and the information detected throughout the open channels, an attacker can quickly determine the session key.
Li et al. [17]	2020	Prone to insider sensor impersonation, impersonation, and ESL attacks; has poor usability.
Sadhukhan et al. [18]	2021	Prone to replay, ESL, man-in-the-middle, and DoS attacks; lacking user anonymity and timely typo detection.
Wazid et al. [19]	2021	Prone to offline password guessing, insider sensor impersonation, desynchronization, and man-in-the-middle attacks; lacking user untraceability and PFS; has high computation.
Srinivas et al. [20]	2021	Prone to session key compromise and device impersonation attacks; lacking user anonymity and PFS; has high computation.
Sutrala et al. [21]	2022	Prone to smart card/device loss and offline password guessing attacks; lacking user untraceability; has high computation.
Tanveer et al. [22]	2022	Prone to man-in-middle, insider sensor impersonation, and offline password guessing attacks; lacking user untraceability and PFS.
Chen et al. [24]	2023	Prone to insider sensor impersonation and ESL attacks; has poor usability.
Wang et al. [25]	2023	Prone to insider IoT device impersonation and ESL attacks.
Kumar et al. [26]	2024	Prone to insider sensor impersonation attacks and ESL attacks; lacking PFS and key privacy.

**Table 2:** Notations of the protocols.

Notation	Description
A	Adversary
$U_{i},ID_{ui},PW_{ui},BIO_{ui}$	User and his/her identity, password, biometric
$S_{j},ID_{sj}$	Sever (register left) and its identity
$S_{k},ID_{sk}$	IoT device and its identity
s/PK	Private keys/public keys
*r*	Random numbers
*T*	Timestamps
SSK	The session key
Gen()/Rep()	Biometric key generation/reproduction algorithm
$\sigma_{ui}$/$\tau_{ui}$	Biometric secret key/public reproduction parameter
et	Fuzzy extractor error tolerance threshold value
$w_{sk},w_{sj}$	Shared secrets of RC and IoT device
$wt_{ui},wt_{sj}$	Shared secrets of RC and user
$T_{ui},L_{ui}$	User dynamic credentials and lists
*x*	Secret values shared by cloud servers and gateways
y,Y	Secret values shared by cloud servers and users
$X_{Gk},x_{Gk}$	Gateway’s secret key
$X_{sk}$	Secret key of IoT device

**Table 3:** Codes for users with ProVerif.

let User=
new dui:bitstring;
let MIDui = Mul (Hash (con (dui, PWui)), P) in
out (schij, (IDui, MIDui));
in (schij, (uxui:bitstring,uPKui: bitstring, uTui: bitstring));
let sui = Add (Hash (con (dui, PWui)), uxui) in
let PKui = Mul (sui, P) in
if PKui = uPKui then
let esui = xor (sui, Hash (con (BIOui, PWui))) in
let LV = Mod (Hash (con (con (con (IDui, PWui), BIOui), sui)), d) in
let wsj = Hash (con (Mul (sui, uPKsj), IDui)) in
!
(
event startAuthSk;
let usui = xor (esui, Hash (con (BIOui, PWui))) in
let uLV = Mod (Hash (con (con (con (IDui, PWui), BIOui), usui)), n) in
if uLV = LV then
new TSSeedui:bitstring;
let T1 = generate_Timeline (TSSeedui) in
let Aui = Mul(rui,PKui) in
let EIDsk = xor (IDsk, Hash (con(con (Aui, Bui),)wsj)) in
let Vui = Hash(con(con(con(con(EIDsk, IDui), Aui), T1), wsj)) in
out (chij, (Aui,uTui, EIDsk, T1, Vui));
in (chki, (sAsk: bitstring,sT3: bitstring,sEIDsk: bitstring,
sTui: bitstring,sVsk:bitstring));
let Sik = Mul (mul (rui, sui), sAsk) in
let uIDsk = xor (sEIDsk, Hash (con(sAsk,Sik))) in
let uTui = Hash (con (xor (sTui, Hash (con(sAsk,Sik))),wsj)) in
let uVsk = Hash (con (con (con(con (uIDsk, IDui),sAsk), Sik), sT3))
in
if uVsk = sVsk then
let SKik = Hash (con (con (Sik,IDui), uIDsk))in
event endAuthUi;
0
).

**Table 4:** Simulation results.

Result	Target
RESULT Weak secret IDui is true (bad not derivable)	Anonymity
RESULT Weak secret IDsK is true (bad not derivable)	Anonymity
RESULT not attacker(sui[]) is true	Long-term secret security
RESULT not attacker(ssj[]) is true	Long-term secret security
RESULT not attacker(ssk[]) is true	Long-term secret security
RESULT not attacker(rui[]) is true	Ephemeral secret security
RESULT not attacker(rsk[]) is true	Ephemeral secret security
RESULT not attacker (SSKik[]) is true	Session key security
RESULT not attacker (SSKki[]) is true	Session key security
RESULT inj-event(endAuthUi) ⇒ inj-event(startAuthUi) is true	Consistent
RESULT inj-event(endAuthSk) ⇒ inj-event(startAuthSk) is true	Consistent

**Table 5:** Security feature comparison.

Scheme	F1	F2	F3	F4	F5	F6	F7	F8	F9	F10	F11	F12	F13	F14	F15
[17]	✓	✓	×	✓	×	✓	×	×	✓	✓	✓	✓	✓	✓	✓
[19]	×	×	×	×	✓	✓	×	×	✓	✓	✓	✓	×	✓	✓
[20]	×	×	×	×	✓	✓	×	×	✓	×	✓	✓	✓	✓	✓
[21]	×	×	✓	×	✓	✓	×	✓	✓	×	✓	✓	✓	✓	✓
[22]	×	✓	✓	×	✓	✓	×	×	✓	✓	✓	✓	×	✓	✓
[25]	✓	✓	✓	✓	×	✓	×	✓	✓	✓	✓	✓	✓	✓	✓
[26]	✓	×	×	✓	×	✓	×	✓	✓	✓	✓	✓	✓	✓	✓
Ours	✓	✓	✓	✓	✓	✓	✓	✓	✓	✓	✓	✓	✓	✓	✓

F1: Anonymity and untraceability; F2: PFS; F3: key privacy; F4: offline password guessing attack resistance; F5: ephemeral secret leakage attack resistance; F6: smart card loss attack resistance; F7: insider impersonation attack resistance; F8: man-in-the-middle attack resistance; F9: replay attack resistance; F10: IoT node capture attacks; F11: password-friendly; F12: timely typo detection; F13: mutual authentication and key agreement; F14: password/biometric update locally; F15: no password authentication table. ✓: supporting a functional feature or ensuring security; ×: lacking a functional feature or not ensuring security.

**Table 6:** Communication costs.

Scheme	User	TA	Sensor	Total (bit)	Total (↓%)
Ours	G + H + TS + 2ID = 928	G + H + TS + ID = 800	G + H + TS + ID = 800	2528	-
[17]	G + H + 2ID = 896	2G + 4H = 1792	G + 2H = 896	3584	29.5%
[19]	G + 3H + TS + 2ID = 1440	G + 3H + 2TS + 3ID = 1600	G + 3H + 2TS + ID = 1344	4384	41.1%
[20]	G + 3H + TS + ID = 1312	G + 2H + TS = 928	G + 2H + TS + ID = 1056	3296	23.3%
[21]	G + 2H + TS + 3ID = 1312	G + 3H + 2TS + ID = 1344	2G + H + 2TS + ID = 1216	3872	34.7%
[22]	G + 2H + TS + 2ID = 1184	2G + H + R + TS + ID = 1440	2G + H + TS = 1056	3680	31.3%
[25]	G + H + R + 2ID = 1152	2G + 2H + R + ID = 1644	G + H = 640	3456	26.8%
[26]	G + H + TS + 2ID = 928	2G + 2H + 2R + 2TS + 2ID = 2122	H + TS = 384	3328	24.0%

↓: reduction in the overhead of our scheme over existing schemes.

**Table 7:** Run times of different cryptographic primitives.

Notation	Cryptographic Primitive	RC	User/Sensor
$T_{em}$	ECC point multiplication	1.774	3.747
$T_{ea}$	ECC point addition	0.026	0.041
$T_{h}$	Hash operation	0.016	0.032
$T_{ae}$	AEGIS	0.033	0.051
$T_{fe}\approx T_{em}$	Fuzzy extractors	1.774	3.747

**Table 8:** Computation costs.

Scheme	User (ms)	RC (ms)	RC (↓%)	Sensor (ms)	Total (ms)	Total (↓%)
Ours	$T_{fe}+2T_{em}+8T_{h}\approx 11.497$	$5T_{h}\approx 0.08$	-	$2T_{em}+5T_{h}\approx 7.654$	19.231	-
[17]	$T_{fe}+3T_{em}+9T_{h}\approx 15.276$	$T_{em}+8T_{h}\approx 1.902$	95.8%	$2T_{em}+4T_{h}\approx 7.622$	24.8	22.5%
[19]	$T_{fe}+5T_{em}+T_{ea}+19T_{h}\approx 23.131$	$5T_{em}+T_{ea}+T_{h}\approx 8.912$	99.1%	$4T_{em}+T_{ea}+12T_{h}\approx 15.413$	47.456	59.5%
[20]	$T_{fe}+5T_{em}+T_{ea}+17T_{h}\approx 23.067$	$2T_{em}+10T_{h}\approx 3.708$	97.8%	$4T_{em}+T_{ea}+8T_{h}\approx 15.285$	42.06	54.3%
[21]	$T_{fe}+5T_{em}+3T_{ea}+16T_{h}\approx 23.117$	$3T_{em}+2T_{ea}+9T_{h}\approx 5.518$	98.6%	$4T_{em}+2T_{ea}+8T_{h}\approx 15.326$	43.961	56.3%
[22]	$3T_{ae}+T_{fe}+3T_{em}+6T_{h}\approx 15.333$	$3T_{ae}+T_{em}+2T_{h}\approx 1.905$	95.8%	$2T_{ae}+2T_{em}+3T_{h}\approx 7.692$	24.93	22.9%
[25]	$3T_{am}+T_{fe}+8T_{h}\approx 15.244$	$T_{em}+10T_{h}\approx 1.934$	95.9%	$2T_{em}+4T_{h}\approx 7.622$	24.8	22.5%
[26]	$3T_{em}+T_{ea}+T_{fe}+11T_{h}\approx 15.381$	$4T_{ae}+T_{ea}+9T_{h}\approx 7.266$	98.9%	$2T_{ae}+6T_{h}\approx 7.686$	30.333	36.6%

↓: reduction in the overhead of our scheme over existing schemes.

## Data Availability

Data are contained within the article. The original contributions presented in this study are included in the article. Further inquiries can be directed to the corresponding authors.

## References

[1] Lin J., Yu W., Zhang N., Yang X., Zhang H., Zhao W. (2017). A Survey on Internet of Things: Architecture, Enabling Technologies, Security and Privacy, and Applications. IEEE Internet Things J..

[2] Wang D., Li W., Wang P. (2018). Measuring Two-Factor Authentication Schemes for Real-Time Data Access in Industrial Wireless Sensor Networks. IEEE Trans. Ind. Inform..

[3] Gope P., Das A.K., Kumar N., Cheng Y. (2019). Lightweight and Physically Secure Anonymous Mutual Authentication Protocol for Real-Time Data Access in Industrial Wireless Sensor Networks. IEEE Trans. Ind. Inform..

[4] Prouff E., Rivain M., Bevan R. (2009). Statistical Analysis of Second Order Differential Power Analysis. IEEE Trans. Comput..

[5] Wang P.J., Zhang Y.J., Zhang X.L. (2012). Research of differential power analysis countermeasures. Dian Zi Yu Xin Xi Xue = Bao J. Electron. Inf. Technol..

[6] Abulencia J. (2021). Insider attacks: Human-factors attacks and mitigation. Comput. Fraud. Secur..

[7] Robayo T.A. (2022). The Enemy Within: A Framework for Understanding the Lifecycle of the Malicious Insider Threat to Information Systems. Ph.D. Thesis.

[8] Wang D., Wang P. (2018). Two Birds with One Stone: Two-Factor Authentication with Security Beyond Conventional Bound. IEEE Trans. Dependable Secur. Comput..

[9] Jiang Q., Ma J., Wei F., Tian Y., Shen J., Yang Y. (2016). An untraceable temporal-credential-based two-factor authentication scheme using ECC for wireless sensor networks. J. Netw. Comput. Appl..

[10] Wu F., Xu L., Kumari S. (2017). A privacy-preserving and provable user authentication scheme for wireless sensor networks based on Internet of Things security. J. Ambient. Intell. Humaniz. Comput..

[11] Chang C.C., Le H.D. (2016). A Provably Secure, Efficient, and Flexible Authentication Scheme for Ad hoc Wireless Sensor Networks. IEEE Trans. Wirel. Commun..

[12] Challa S., Wazid M., Das A.K., Kumar N., Goutham Reddy A., Yoon E.J., Yoo K.Y. (2017). Secure Signature-Based Authenticated Key Establishment Scheme for Future IoT Applications. IEEE Access.

[13] Li X., Niu J., Bhuiyan M.Z.A., Wu F., Karuppiah M., Kumari S. (2018). A Robust ECC-Based Provable Secure Authentication Protocol with Privacy Preserving for Industrial Internet of Things. IEEE Trans. Ind. Inform..

[14] Li X., Niu J., Kumari S., Wu F., Sangaiah A.K., Choo K.K.R. (2018). A three-factor anonymous authentication scheme for wireless sensor networks in internet of things environments. J. Netw. Comput. Appl..

[15] Shuai M., Yu N., Wang H., Xiong L. (2019). Anonymous authentication scheme for smart home environment with provable security. Comput. Secur..

[16] Yeh H.L., Chen T.H., Liu P.C., Kim T.H., Wei H.W. (2011). A Secured Authentication Protocol for Wireless Sensor Networks Using Elliptic Curves Cryptography. Sensors.

[17] Li X., Peng J., Obaidat M.S., Wu F., Khan M.K., Chen C. (2020). A Secure Three-Factor User Authentication Protocol with Forward Secrecy for Wireless Medical Sensor Network Systems. IEEE Syst. J..

[18] Sadhukhan D., Ray S., Biswas G.P., Khan M.K., Dasgupta M. (2021). A lightweight remote user authentication scheme for IoT communication using elliptic curve cryptography. J. Supercomput..

[19] Wazid M., Das A.K., Kumar N., Alazab M. (2021). Designing Authenticated Key Management Scheme in 6G-Enabled Network in a Box Deployed for Industrial Applications. IEEE Trans. Ind. Inform..

[20] Srinivas J., Das A.K., Wazid M., Vasilakos A.V. (2021). Designing Secure User Authentication Protocol for Big Data Collection in IoT-Based Intelligent Transportation System. IEEE Internet Things J..

[21] Sutrala A.K., Obaidat M.S., Saha S., Das A.K., Alazab M., Park Y. (2022). Authenticated Key Agreement Scheme with User Anonymity and Untraceability for 5G-Enabled Softwarized Industrial Cyber-Physical Systems. IEEE Trans. Intell. Transp. Syst..

[22] Tanveer M., Khan A.U., Kumar N., Hassan M.M. (2022). RAMP-IoD: A Robust Authenticated Key Management Protocol for the Internet of Drones. IEEE Internet Things J..

[23] Wang Q., Wang D. (2023). Understanding Failures in Security Proofs of Multi-Factor Authentication for Mobile Devices. IEEE Trans. Inf. Forensics Secur..

[24] Chen C., Guo H., Wu Y., Gao Y., Liu J. (2023). A novel two-factor multi-gateway authentication protocol for WSNs. Ad Hoc Netw..

[25] Wang C., Wang D., Duan Y., Tao X. (2023). Secure and Lightweight User Authentication Scheme for Cloud-Assisted Internet of Things. IEEE Trans. Inf. Forensics Secur..

[26] Kumar C.M., Dwivedi S.K., Brindha M., Al-Shehari T., Alfakih T., Alsalman H., Amin R. (2024). REPACA: Robust ECC based privacy-controlled mutual authentication and session key sharing protocol in coalmines application with provable security. Peer-to-Peer Netw. Appl..

[27] Krawczyk H. (2005). HMQV: A high-performance secure diffie-hellman protocol. Advances in Cryptology—CRYPTO 2005: 25th Annual International Cryptology Conference, Santa Barbara, CA, USA, 14–18 August 2005.

[28] Lu Y., Xu G., Li L., Yang Y. (2019). Anonymous three-factor authenticated key agreement for wireless sensor networks. Wirel. Netw..

[29] Dolev D., Yao A. (1983). On the security of public key protocols. IEEE Trans. Inf. Theory.

[30] LaMacchia B., Lauter K., Mityagin A. (2007). Stronger security of authenticated key exchange. Provable Security: First International Conference, ProvSec 2007, Wollongong, Australia, 1–2 November 2007.

[31] Yoneyama K. (2009). Efficient and strongly secure password-based server aided key exchange. Inf. Media Technol..

[32] Wang D., He D., Wang P., Chu C.H. (2014). Anonymous two-factor authentication in distributed systems: Certain goals are beyond attainment. IEEE Trans. Dependable Secur. Comput..

[33] Das M.L. (2009). Two-factor user authentication in wireless sensor networks. IEEE Trans. Wirel. Commun..

[34] Gope P., Hwang T. (2016). A Realistic Lightweight Anonymous Authentication Protocol for Securing Real-Time Application Data Access in Wireless Sensor Networks. IEEE Trans. Ind. Electron..

[35] Jiang Q., Zeadally S., Ma J., He D. (2017). Lightweight three-factor authentication and key agreement protocol for internet-integrated wireless sensor networks. IEEE Access.

[36] Subramani J., Maria A., Rajasekaran A.S., Al-Turjman F. (2022). Lightweight Privacy and Confidentiality Preserving Anonymous Authentication Scheme for WBANs. IEEE Trans. Ind. Inform..

[37] Gope P., Sikdar B. (2020). An Efficient Privacy-Preserving Authenticated Key Agreement Scheme for Edge-Assisted Internet of Drones. IEEE Trans. Veh. Technol..

[38] Halevi S., Krawczyk H. (1999). Public-Key Cryptography and Password Protocols. ACM Trans. Inf. Syst. Secur..

[39] Wang D., Wang P. (2014). Understanding security failures of two-factor authentication schemes for real-time applications in hierarchical wireless sensor networks. Ad Hoc Netw..

[40] Hankerson D., Menezes A.J., Vanstone S. (2003). Guide to Elliptic Curve Cryptography.

[41] Gura N., Patel A., Wander A., Eberle H., Shantz S.C. (2004). Comparing Elliptic Curve Cryptography and RSA on 8-bit CPUs. Cryptographic Hardware and Embedded Systems—CHES 2004: 6th International Workshop Cambridge, MA, USA, 11–13 August 2004.

[42] Srinivas J., Das A.K., Li X., Khan M.K., Jo M. (2021). Designing Anonymous Signature-Based Authenticated Key Exchange Scheme for Internet of Things-Enabled Smart Grid Systems. IEEE Trans. Ind. Inform..

[43] Guo Y., Guo Y. (2023). CS-LAKA: A Lightweight Authenticated Key Agreement Protocol with Critical Security Properties for IoT Environments. IEEE Trans. Serv. Comput..

[44] Wang P., Li B., Shi H., Shen Y., Wang D., Li F. (2019). Revisiting Anonymous Two-Factor Authentication Schemes for IoT-Enabled Devices in Cloud Computing Environments. Secur. Commun. Netw..

